# Healthcare utilization and expenditures among adults with type 2 diabetes mellitus and comorbid psychological distress

**DOI:** 10.3389/fendo.2026.1702996

**Published:** 2026-01-29

**Authors:** Wenjie Qu, Ying Wang, Yaqin Zhong, Xin Cao, Yuting Wang, Rongrong Chen, Miaomiao Zhao, Weiping Yang, Yuexia Gao

**Affiliations:** 1 Department of Health Management, School of Public Health, Nantong University, Jiangsu, China; 2 Institute for Health Development, Nantong University, Jiangsu, China; 3 Department of Neurosurgery, Medicine College, Nantong University, Jiangsu, China; 4 Department of Neurosurgery, Affiliated Hospital of Nantong University, Jiangsu, China; 5 Department of Chronic Diseases, Dafeng People’s Hospital, Yancheng, China

**Keywords:** depressive symptoms, diabetes distress, health services use, healthcare costs, rural population

## Abstract

**Background:**

Depressive symptoms and diabetes distress are associated with adverse outcomes in adults with type 2 diabetes mellitus (T2DM). However, no prior studies evaluated the association between healthcare expenditures, utilization, and psychological health in adults with T2DM in rural areas of China. The aim of this study was to explore the association between psychological health—specifically depressive symptoms and diabetes distress—and healthcare utilization and expenditures in this population.

**Methods:**

This cross-sectional study was conducted in 15 rural health clinics in Jiangsu Province, China, and involved 843 adults with T2DM. Psychological health was assessed using the 17-item Diabetes Distress Scale (DDS-17) and the 10-item Center for Epidemiologic Studies Depression Scale (CESD-10). Healthcare utilization and expenditures were evaluated with negative binomial, logistic, and ordinary least squares (OLS) regression models.

**Results:**

Of 843 participants, a total of 32.62% of the sample were comorbid with distress, depression, or both. Each 1-point increase in diabetes distress score was associated with a 44% increase in the expected number of annual outpatient visits (IRR = 1.44, P<0.05), a 52% increase in the odds of inpatient service utilization (OR = 1.52, P <0.05), and a 43.3% increase in outpatient costs (e^β^-1 = 0.433, P<0.05). For each point increase in depressive symptom score, the expected number of annual outpatient visits increased by 4% (IRR = 1.04, P<0.01), the odds of inpatient service utilization increased by 4% (OR = 1.04, P <0.01), and total medical expenditures increased by 2% (e^β^-1 = 0.02, P<0.05).

**Conclusion:**

Our study observed that Diabetes distress is associated with higher outpatient costs, while diabetes with depressive symptoms is associated with higher total medical expenditures. These findings suggest that psychological screening and care interventions for adults with diabetes may be essential.

## 1 Introduction

Diabetes mellitus (DM) is a complex chronic disease that is one of the most important public health problems due to its high prevalence and cost of healthcare (1). DM and its complications represent a huge burden on healthcare service utilization and expenditure, resulting in global healthcare expenditures of 966 billion USD in 2021 (1–3). Diabetes often combines with other complications, such as diabetic nephropathy (4), cardiomyopathy (5), retinopathy (6), osteoporosis (7), and psychiatric symptoms (8). Depression and diabetes distress are two major forms of psychological distress frequently experienced by adults with type 2 diabetes mellitus (T2DM), increasing the risk of mortality, diabetes-related complications, and poor self-management and quality of life (9, 10). According to systematic review studies, the overall estimated prevalence of depression in people with T2DM is 19%-28% (11, 12). Studies in the U.S., Canada, Denmark, and Bangladesh reported that diabetes distress affects about one-third of adults with T2DM (13–16). In addition, depression and diabetes distress can also overlap, with approximately 4.5% of adults with diabetes screening positive for both (17). Depression and diabetes intersect in pathogenesis, such as inflammation (18, 19), oxidative stress (20, 21), metabolic disorders (18, 22), and others. Moreover, the relationship between psychological health and T2DM can be bidirectional (23–25). Therefore, depression and diabetes distress may lead to poorer glycemic control. The economic burden of diabetes, especially comorbid psychological and emotional disorders, deserves further study.

The increased prevalence rates of comorbid psychological distress raise the issue of the association of psychological distress and healthcare expenditures and utilization (26). Studies based on the National Health Insurance Research Database (NHIRD) from Taiwan revealed that healthcare utilization and expenditures for persons with DM comorbid mental disorders were significantly higher than non-comorbid individuals (8, 27). Additionally, one study with 400,495 individuals with DM in the United States found that major depressive disorder contributed to an increase in the excess medical expenditures among persons with diabetes by increasing inpatient care, outpatient care, and medication expenditures (28). Another recent population-based cohort study in Canada suggested that comorbid mental disorders can lead to higher healthcare costs for persons with chronic diseases through increased hospitalization, emergency department visits, and length of hospital stay (29). However, studies on diabetes, mental health, and health care spending and utilization are inconsistent. A previous cross-sectional study in the German National Health Interview and Examination Survey (GNHIES) indicated no increase in the length of hospitalization or visits to mental health specialists among adults with DM comorbid mental disorders (30). Meanwhile, fewer prior studies have examined the healthcare costs associated with psychological distress in Chinese adults with T2DM. Additionally, less attention has been paid to differences in various psychological distresses. Hence, the association of psychological distress with healthcare expenditures and utilization in adults with T2DM in the context of the increasing prevalence of co-existing diabetes and psychological distress was worthy of further study.

Structured interviews for the clinical diagnosis of depression are impractical in large-scale surveys (31). The definition of depression is based on symptoms forming a syndrome and causing functional impairment (32). This study aimed to estimate the healthcare expenditures and healthcare utilization associated with depressive symptoms (measured by the CESD-10) and diabetes distress (measured by the DDS-17) in Chinese adults with T2DM. Specifically, the following research questions will be addressed: (I) To what extent are comorbid depressive symptoms associated with increased healthcare expenditures and utilization in adults with T2DM? (II) To what extent is comorbid diabetes distress associated with increased healthcare expenditures and utilization in persons with T2DM? (III) How do the magnitude and pattern of associations with healthcare utilization and expenditures differ between diabetes distress and depressive symptoms? (IV) Do these associations persist after adjusting for key demographic characteristics?

## 2 Materials and methods

### 2.1 Study design and participants

We designed a cross-sectional study at baseline in 15 rural health clinics of Jiangsu Province in southeastern China. The study sample consisted of 5 health clinics located in rural communities in Tongzhou district, Nantong city, and 10 health clinics in Dafeng district, Yancheng city. Participants with type2 diabetes were randomly drawn from physician-managed registries. Data were collected through face-to-face, interviewer -assisted questionnaires administered on-site at the community health centers by trained local surveyors. From January to March 2019, we identified from recruited adults who (I) had been diagnosed with T2DM for at least 1 year by physicians; (II) were aged 45 years or above; and (III) were able to communicate in Mandarin. We excluded adults with severe diabetes complications or functional deficits, schizophrenia, severe dementia, or bipolar disorder, and a traumatic experience in the past 6 months. Eventually, 900 individuals from 15 clinics participated in the study, and 843 valid questionnaires were collected, with a response rate of 93.67%.

### 2.2 Measures

#### 2.2.1 Predictor variables

##### 2.2.1.1 Diabetes distress

Diabetes distress was assessed with the 17-item Diabetes Distress Scale (DDS-17) (33). Participants rated the degree to which they felt the social and medical care distress related to diabetes and its management. Item responses were based on a Likert scale ranging from 1 (not a problem) to 6 (a severe problem), with higher total scores reflecting greater diabetes distress. A mean score ≥2 serves as the cut-point for moderate or greater diabetes distress, indicating a level of distress worthy of clinical attention (34). The DDS-17 was shown to have good psychometric properties for internal consistency reliability (α=0.90) and test-retest reliability coefficient (α=0.74) in Chinese adults with type 2 diabetes mellitus (35). The DDS-17 demonstrated good reliability in the present study (α = 0.87).

##### 2.2.1.2 Depressive symptoms

Depressive symptoms were assessed with the short form 10-item Center for Epidemiologic Studies Depression Scale (CESD-10) (36). It shows the extent to which individuals experienced depressive symptoms during the prior week and has been validated as a tool to identify individuals at high risk of depression with wide applicability in the general population. The CESD-10 was self-rated by T2DM adults on a 4-point Likert scale, where 0 = “rarely or none during the time (<1 day)” to 3 = “most of the time (5–7 days).” The scores of the CESD-10 range from 1 to 30, with higher scores indicating more severe depressive symptoms, and individuals who had scores≥10 were classified as having high depressive symptoms. The CESD-10 has high reliability and validity in Chinese middle-aged and elderly populations (α = 0.82) (37). Cronbach’s ɑ=0.85 indicated that the scale was reliable.

#### 2.2.2 Dependent variables

##### 2.2.2.1 Healthcare utilization

Healthcare service utilization was measured as the annual number of outpatient visits (preventive, acute care, and emergency department) and the utilization of inpatient services and length of stay per year by a self-designed questionnaire. Preventive visits included general check-ups and visits for immunizations. Acute visits included all other outpatient visits not categorized as preventive. The participants were asked to answer three questions regarding their status of healthcare utilization: “Have you had a doctor visit or other healthcare professional in the past year?” “Have you had any outpatient visits or emergency department visits in the past year, including preventive and acute care?” and “Have you been hospitalized for any length of time in the past year, and if so, report your length of stay?”

##### 2.2.2.2 Healthcare expenditures

The healthcare expenditure refers to all direct payments made for healthcare services and products within a calendar year, including costs for outpatient services, inpatient services, and drugs. Three expenditure outcomes were assessed: outpatient costs, drug costs, and total medical expenditures. Total medical expenditures summed outpatient, inpatient, and drug costs incurred for the calendar year. The costs associated with primary, specialty, and ancillary care visits were included in the outpatient costs. Inpatient costs comprised those related to admissions with overnight stays, admissions without overnight stays, and emergency department visits that occurred right before an admission. Drug costs included prescription medicines, over-the-counter medicines, medical devices, and dietary supplements. Expenditure data were based primarily on participant self-reports. For costs that were difficult to recall precisely, some participants supplemented their recall by presenting electronic or paper fee statements from healthcare providers. Participants were instructed to report the total amount paid for healthcare, which included both third-party-payer payments and out-of-pocket payments.

#### 2.2.3 Control variables

Covariates in this study include demographic characteristics: gender (male and female), age group (<60, 60-70, and >70), education (illiterate, primary school, secondary school, post-secondary school), marital status (married/unmarried), and features involving clinical diabetes: duration of diabetes and comorbidities. Comorbidities were based on self-reported diagnoses in the past 12 months, including cardiovascular diseases, hypertension, cerebrovascular diseases, and other chronic conditions.

### 2.3 Statistical analyses

Statistical analyses were conducted with STATA software version 16. In the main analyses, the psychological variables were treated as continuous. Descriptive statistics with t-tests and χ2 tests were used to compare differences in the study sample of psychological variables in continuous forms (**Table 1**). Then, multivariable regression models with negative binomial distributions were used to examine the association of psychological variables with the annual number of outpatient visits, and logistic regression models were used to explore the utilization of inpatient services in adults with T2DM (**Table 2**). Model 1,3 only explored the relationship between psychological variable scores and the annual number of outpatient visits and utilization of inpatient services. Model 2,4 further examined the relationship between psychological variables scores and outpatient and inpatient utilization by adding demographic characteristics. The ordinary least squares (OLS) regression models and log links were used to estimate the association of psychological variables with healthcare expenditures, including outpatient costs, drug costs, inpatient costs, and total medical expenditures (**Table 3**). Model 1,3,5 only explored the relationship between psychological variable scores and healthcare expenditures. Model 2,4,6 further examined the relationship between psychological variables scores and healthcare expenditures by adding demographic characteristics. Age, gender, education, marital status, duration of diabetes, chronic medical conditions, and baseline fasting plasma glucose level are covariates, and they are controlled for in the models. All statistical tests were two-tailed, with α set to 0.05. We tested the collinearity issues, and VIF showed that there was no such issue (VIF<5) (38).

**Table 1:** Basic characteristics in adults with type 2 diabetes mellitus (N = 843).

Variable	Mean ± SD/M± QR/n (%)	Diabetes distress score	P	Depressive symptoms score	p
Gender					
Male	282 (33.45)	1.41 ± 0.40	0.001†	5.15 ± 5.79	< 0.001‡
Female	561 (66.55)	1.51 ± 0.45		6.98 ± 5.88	
Age (year)	66.08 ± 9.10				
<60	199 (23.61)	1.50 ± 0.44	0.019*	5.53 ± 5.52	0.005†
60-70	328 (38.91)	1.52 ± 0.47		7.15 ± 6.26	
> 70	316 (37.94)	1.42 ± 0.41		6.09 ± 5.70	
Education					
Illiterate	332 (39.38)	1.53 ± 0.45	0.005†	7.37 ± 5.99	< 0.001‡
Primary	252 (29.89)	1.48 ± 0.43		6.95 ± 6.21	
Secondary	190 (22.54)	1.43 ± 0.45		4.59 ± 5.00	
Post-Secondary	69 (8.19)	1.35 ± 0.34		4.33 ± 5.21	
Marital status					
Married	695 (82.44)	1.48 ± 0.44	0.947	7.17 ± 6.17	0.070
Unmarried^a^	148 (17.56)	1.48 ± 0.44		6.2 ± 5.85	
Comorbidities^b^					
Cardiovascular diseases	135 (16.01)	1.50 ± 0.43	0.505	6.98 ± 5.72	1.192
Hypertension	490 (58.13)	1.49 ± 0.43	0.444	6.57 ± 5.91	0.259
Cerebrovascular diseases	72 (8.54)	1.58 ± 0.41	0.030*	8.82 ± 6.62	< 0.001‡
Other diseases	432 (51.25)	1.53 ± 0.47	0.001†	6.98 ± 6.16	0.002†
Baseline FPG (mmol/L)	8.16 ± 2.51				
FPG≥7.0	524 (62.16)	1.47 ± 0.43	0.7.09	6.39 ± 6.06	0.904
FPG<7.0	319 (37.84)	1.48 ± 0.45		6.34 ± 5.67	
Duration of diabetes	8.56 ± 5.76				
Diabetes distress scores	1.48 ± 0.44				
Moderate or severe distress (DDS≥2)	105 (12.46)	-	-	-	-
Depression scores	6.37 ± 5.91				
High Depressive Symptoms(CESD≥10)	212 (25.15)	-	-	-	-
Utilization of medical services					
Number of outpatient visits per year	0 ± 2	-	-	-	-
Utilization of inpatient services	249 (29.54)	-	-	-	-
Healthcare expenditures(CNY)					
Outpatient costs	700 ± 1700	-	-	-	-
Drug costs	1000 ± 2100	-	-	-	-
Inpatient costs	6000 ± 10500	-	-	-	-
Total medical expenditures	3500 ± 7950	-	-	-	-

DDS, Diabetes Distress Scale; CES-D, Center for Epidemiologic Studies Depression Scale; FPG, fasting plasma glucose; CNY, Chinese Yuan.

a unmarried included separated, divorced, widowed, or never married.

b Comorbidities included cardiovascular diseases, hypertension, cerebrovascular diseases, and other chronic conditions.

*P < 0.05; † P < 0.01;‡ P < 0.001.

**Table 2:** Regression analysis of psychological health on healthcare utilization in adults with type 2 diabetes mellitus.

Variable	Group	Model 1	Model 2	Model 3	Model 4
		IRR/OR (95%CI)	IRR/OR (95%CI)	IRR/OR (95%CI)	IRR/OR (95%CI)
Annual number of outpatient visits					
DDS scores		1.52 (1.10-2.10)*	1.44 (1.05-1.98)*		
CES-D scores				1.04 (1.01-1.06)†	1.04 (1.02-1.07)†
Age			0.98 (0.96-0.99)*		0.98 (0.96-1.00)*
Gender (ref: Male)	Female		0.87 (0.60-1.25)		0.84 (0.58-1.20)
Education (ref: illiterate)	Primary		0.85 (0.57-1.26)		0.82 (0.56-1.21)
	Secondary		0.91 (0.58-1.43)		1.05 (0.67-1.65)
	Post-Secondary		0.94 (0.48-1.84)		1.01 (0.51-1.99)
Marital status (ref: Unmarried)	Married		1.42 (0.93-2.17)		1.43 (0.94-2.18)
Duration of diabetes			1.02 (1.00-1.05)		1.02 (00.99-1.05)
Cardiovascular disease (ref: Without)	With		1.68 (1.13-2.50)*		1.70 (1.15-2.52)*
Hypertension (ref: Without)	With		1.26 (0.92-1.72)		1.28 (0.94-1.76)
cerebrovascular disease (ref: Without)	With		1.88 (1.12-3.18)*		1.95 (1.16-3.27)*
With other chronic diseases (ref: Without)	With		1.61 (1.20-2.17)†		1.70 (1.27-2.29)‡
Baseline FPG			0.98 (0.93-1.04)		0.97 (0.92-1.03)
Utilization of inpatient services					
DDS scores		1.61 (1.16-2.23)†	1.52 (1.08-2.15)*		
CES-D scores				1.05 (1.03-1.08)‡	1.04 (1.01-1.07)†
Age			1.02 (1.00-1.04)		1.02 (1.00-1.34)
Gender (ref: Male)	Female		0.71 (0.49-1.02)		0.70 (0.48-1.01)
Education (ref: illiterate)	Primary		1.07 (0.73-1.57)		1.06 (0.72-1.55)
	Secondary		0.88 (0.55-1.41)		0.93 (0.58-1.49)
	Post-Secondary		0.71 (0.35-1.44)		0.72 (0.36-1.45)
Marital status (ref: Unmarried)	Married		1.14 (0.75-1.73)		1.17 (0.77-1.78)
Duration of diabetes			1.04 (1.01-1.07)†		1.04 (1.01-1.07)†
Cardiovascular disease (ref: Without)	With		1.19 (0.79-1.79)		1.19 (0.79-1.80)
Hypertension (ref: Without)	With		1.11 (0.80-1.54)		1.12 (0.81-1.55)
cerebrovascular disease (ref: Without)	With		2.03 (1.22-3.37)†		1.94 (1.16-3.24)*
With other chronic diseases (ref: Without)	With		1.69 (1.23-2.33)†		1.70 (1.23-2.34)†
Baseline FPG			0.99 (0.93-1.05)		0.98 (0.92-1.05)

All coefficients are unstandardized. Gender was coded as male=0, female=1. Education was coded as illiterate=0, primary=1, secondary=2, post-secondary=3. Marital status was coded as unmarried=0, married=1. Cardiovascular disease was coded as without=0, with=1. Hypertension was coded as without=0, with=1. Cerebrovascular disease was coded as without=0, with=1. Other chronic conditions were coded as without=0, with=1. Utilization of inpatient services was coded as without=0, with=1.

DDS, Diabetes Distress Scale; CES-D, Center for Epidemiologic Studies Depression Scale; FPG, fasting plasma glucose; IRR, incidence rate ratio.

*P < 0.05; †P < 0.01; ‡P < 0.001.

**Table 3:** Regression analysis of distress and depressive symptoms on healthcare expenditures in adults with type 2 diabetes mellitus.

Variable	Group	Outpatient costs		Drug costs		Total medical expenditures	
		Model 1	Model 2	Model 3	Model 4	Model 5	Model 6
		β (95%CI)	β (95%CI)	β (95%CI)	β (95%CI)	β (95%CI)	β (95%CI)
Diabetes distress							
DDS scores		0.35 (0.02-0.67)*	0.36 (0.03-0.68)*	0.13 (-0.28-0.54)	0.19 (-0.24-0.62)	0.23 (-0.06-0.52)	0.26 (-0.04-0.56)
CES-D scores							
Age			0.01 (-0.02-0.02)		0.01 (-0.02-0.03)		0.01 (-0.01-0.03)
Gender (ref: Male)	Female		-0.02 (0.39-0.35)		0.14 (-0.32-0.60)		-0.15 (-0.48-0.18)
Education (ref: illiterate)	Primary		-0.10 (-0.49-0.29)		0.12 (-0.36-0.59)		0.21 (-0.14-0.56)
	Secondary		0.42 (-0.03-0.86)		0.22 (-0.32-0.77)		0.04 -0.38-0.46)
	Post-Secondary		0.16 (-0.48-0.80)		0.11 (-0.64-0.86)		0.08 (-0.54-0.69)
Marital status (ref: Unmarried)	Married		0.02 (-0.41-0.44)		0.19 (-0.34-0.73)		0.08 (-0.30-0.45)
Duration of diabetes			0.03 (-0.00-0.15)		0.03 (0.01-0.06)*		0.02 (-0.01-0.04)
Cardiovascular disease (ref: Without)	With		0.21 (-0.17-0.59)		-0.25 (-0.72-0.23)		0.07 (-0.29-0.43)
Hypertension (ref: Without)	With		0.06 (-0.26-0.38)		0.20 (-0.19-0.59)		0.02 (-0.27-0.32)
cerebrovascular disease (ref: Without)	With		0.08 (-0.39-0.55)		-0.23 (-0.79-0.33)		0.24 (-0.20-0.69)
With other chronic diseases (ref: Without)	With		0.03 (-0.29-0.35)		-0.08 (-0.46-0.31)		-0.01 (-0.29-0.28)
Baseline FPG			-0.05 (-0.11-0.01)		-0.01 (-0.09-0.06)		-0.02 (-0.07-0.04)
Depressive symptoms							
DDS scores							
CES-D scores		0.02 (-0.00-0.04)	0.02 (-0.01-0.05)	0.02 (-0.01-0.05)	0.02 (-0.01-0.05)	0.03 (0.01-0.05)*	0.02 (0.01-0.05)*
Age			0.01 (-0.02-0.02)		0.01 (-0.02-0.03)		0.01 (-0.01-0.03)
Gender (ref: Male)	Female		-0.02 (-0.39-0.35)		0.14 (-0.32-0.60)		-0.15 (-0.48-0.18)
Education (ref: illiterate)	Primary		-0.09 (-0.48-0.29)		0.15 (-0.33-0.62)		0.19 (-0.16-0.54)
	Secondary		0.46 (0.01-0.91)*		0.29 (-0.27-0.84)		0.08 (-0.34-0.51)
	Post-Secondary		0.15 (-0.49-0.79)		0.14 (-0.61-0.89)		0.09 (-0.52-0.70)
Marital status (ref: Unmarried)	Married		0.04 (-0.39-0.46)		0.19 (-0.34-0.72)		0.09 (-0.29-0.46)
Duration of diabetes			0.02 (-0.01-0.05)		0.03 (-0.01-0.06)		0.02 (-0.01-0.04)
Cardiovascular disease (ref: Without)	With		0.21 (-0.16-0.59)		-0.24 (-0.72-0.24)		0.08 (-0.28-0.44)
Hypertension (ref: Without)	With		0.07 (-0.26-0.39)		0.19 (-0.20-0.58)		0.03 (-0.27-0.32)
DDS scores							
cerebrovascular disease (ref: Without)	With		0.09 (-0.38-0.56)		-0.22 (-0.77-0.34)		0.23 (-0.22-0.67)
With other chronic diseases (ref: Without)	With		0.06 (-0.26-0.37)		-0.07 (-0.45-0.31)		0.01 (-0.27-0.30)
Baseline FPG			-0.06 (-0.12-0.01)		-0.02 (-0.09-0.06)		-0.02 (-0.07-0.04)

All coefficients are unstandardized. Gender was coded as male=0, female=1. Education was coded as illiterate=0, primary=1, secondary=2, post-secondary=3. Marital status was coded as unmarried=0, married=1. Cardiovascular disease was coded as without=0, with=1. Hypertension was coded as without=0, with=1. Cerebrovascular disease was coded as without=0, with=1. Other chronic conditions were coded as without=0, with=1.

DDS, Diabetes Distress Scale; CES-D, Center for Epidemiologic Studies Depression Scale; FPG, fasting plasma glucose.

*P < 0.05; †P < 0.01; ‡P < 0.001.

In additional analyses, we re-coded the psychological scale scores into categorical variables based on established clinical cut-offs. This created four mutually exclusive groups: (1) Diabetes mellitus (DDS<2, CESD<10), (2) Comorbid diabetes distress (DDS<2, CESD<10), (3) Comorbid depressive symptoms (DDS<2, CESD≥10), and (4) Comorbid diabetes distress and depressive symptoms (DDS≥2, CESD≥10). The same models were applied to the categorical variables to estimate the association of each group relative to the reference group with the outcomes.

## 3 Results

### 3.1 Participants characteristics

Of 843 participants with T2DM, the mean age was 66.8 ± 9.10 years, and 66.55% were women, and the majority(82.44%) were married (**Table 1**). 60.62% had primary education or higher. 74.97% had T2DM for more than 5 years. Hypertension (58.13%) was the most common comorbidity. The average scores of diabetes distress and depressive symptoms were 1.48 (SD 0.44) and 6.37 (SD 5.91), respectively. The rate of inpatient service utilization was 29.54%. The median outpatient cost, median drug cost, median inpatient cost, and median total expenditure were ￥700, ￥1000, ￥6000, and ￥3500, respectively.

### 3.2 Association of psychological distress with healthcare utilization

**Table 2** presented the results of the regression analysis of psychological health on annual number of outpatient visits and utilization of inpatient services. Model 1 showed that for each 1-point increase in the diabetes distress score, the expected number of annual outpatient visits increased by 52% (incidence rate ratio [IRR]=1.52, 95%CI: 1.10-2.10), and the odds of inpatient service utilization increased by 61%(OR = 1.61, 95%CI:1.16-2.23). Model 2, after adjusting for demographic characteristics, showed that the significant association persisted: for every 1-point increase in the diabetes distress score, the expected number of annual outpatient visits increased by 44%(IRR = 1.44, 95%CI: 1.05-1.98), and the odds of inpatient service utilization increased by 52%(OR = 1.52, 95%CI: 1.08-2.15). In Model 3, for each 1-point increase in depressive symptom score, the expected number of annual outpatient visits increased by 4% (IRR = 1.04, 95%CI: 1.01-1.06), and the odds of inpatient service utilization increased by 5% (OR = 1.05, 95%CI: 1.03-1.08). In Model 4, after adjusting for demographic characteristics, the significant association persisted: for each 1-point increase in the depressive symptoms score, the expected number of annual outpatient visits increased by 4% (IRR = 1.04, 95%CI: 1.02-1.07), and the odds of inpatient service utilization increased by 4% (OR = 1.04, 95%CI: 1.01-1.07). Age, comorbid cardiovascular disease, cerebrovascular disease, and other chronic conditions in patients with T2DM were significantly associated with the number of outpatient visits. The duration of disease, comorbid cerebrovascular disease, and other chronic conditions in adults with T2DM were significantly associated with their utilization of inpatient services.

### 3.3 Association of psychological distress with healthcare expenditures

Results from the ordinary least squares regression were presented in **Table 3**. Outpatient costs were positively associated with diabetes distress scores (p<0.05). Model 1 indicated that each 1-point increase in the diabetes distress score was associated with a 41.9% increase in outpatient costs (e^β^-1 = 0.419, 95%CI: 0.02-0.67). Model 2 indicated that each 1-point increase in diabetes distress score was associated with a 43.3% increase in outpatient costs (e^β^-1 = 0.433, 95%CI: 0.03-0.68). Drug costs, inpatient costs, and total medical expenditures were not related to psychological distress scores (P>0.05), and the duration of disease in adults with T2DM was a factor affecting drug costs.

The total medical expenditures were positively associated with depressive symptom scores (p<0.05). In Model 5, a 3% increase in outpatient costs (e^β^-1 = 0.03, 95%CI: 0.01-0.05) was observed for each 1-point increase in a depressive symptom score. In Model 6, each 1-point increase in depressive symptom score was associated with a 2% increase in total medical expenditures (e^β^-1 = 0.02, 95%CI: 0.01-0.05). Drug costs, inpatient costs, and total medical expenditures were not associated with depressive symptom scores (P>0.05). The education level was one of the factors that influenced outpatient costs.

### 3.4 Additional analysis

Results from sensitivity analyses further verified the robustness of the main analysis results (**Additional Tables 3, 4**). Sensitivity analysis showed that there were significant differences in age, sex, education level, marital status, duration of diabetes, distress score, depressive symptom score, annual number of outpatient visits, inpatient service utilization, outpatient costs, and total medical expenditures (P<0.05) among the four groups (**Additional Tables 1, 2**). The total medical expenditures were higher in adults with psychological health issues compared to the group of adults with T2DM (**Additional Table 4**). A total of 32.62% of the participants were comorbid with distress, depressive symptoms, or both(CESD ≥10 and/or DDS ≥2) (**Additional Figure 1**).

## 4 Discussion

To our knowledge, this is the first study of the association of T2DM healthcare expenditures with diabetes distress and depressive symptoms in rural communities in China. Our study has several findings. First, diabetes distress and depressive symptoms were associated with an increased expected number of annual outpatient visits and inpatient service utilization. Second, diabetes distress was associated with higher outpatient costs, and depressive symptoms were associated with higher total medical expenditures. Third, the duration of the disease factor was associated with drug costs. The education level factor was associated with outpatient costs. Substantial evidence has demonstrated that comorbid depression and depressive symptoms among adults with diabetes was associated with poor diabetes outcomes (39). Mental health issues in adults with diabetes deserve further attention.

The main findings of our study illustrate that poor mental health status was associated with higher expected annual outpatient visits and healthcare utilization in adults with T2DM. Previous studies have also shown that diabetic comorbidities of depressive symptoms or mental distress were associated with higher health service utilization, particularly inpatient care, outpatient visits, and prescriptions (8, 41). Notably, the strength of this association differs between diabetes distress and depressive symptoms. While they overlap to some extent, yet the two concepts are not interchangeable and should be treated as distinct conditions (42). Diabetes distress, as assessed by the DDS-17, refers specifically to the emotional burdens directly related to diabetes management (43, 44). Each one-point increase in its score often has been associated with greater concerns regarding complications, self-management failures, and related issues (34), which may coincide with more frequent healthcare-seeking for diabetes-specific problems. In contrast, depressive symptoms, measured by the CESD-10, encompass a broader range of affective and somatic symptoms; their relatively smaller effect sizes could also be explained if they shape decisions for care indirectly, for instance, by reducing treatment satisfaction (45–47). Similar to previous studies (8, 48), In our study, cardiovascular disease, cerebrovascular disease, and other chronic diseases co-morbidities were positively associated with higher annual outpatient visits and inpatient service utilization. In addition, the duration of T2DM was positively associated with the utilization of inpatient services. People with long-term diabetes are more concerned about their health and more cautious about changes in their condition, resulting in more use of inpatient services. Therefore, further classification for diabetic population for early screening and intervention is needed (49), such as T2DM patients with longer disease duration, multiple comorbidities,and co-existing mental health conditions.

Another meaningful finding of our study is that diabetes distress and depressive symptoms were also linked to higher healthcare expenditures for adults with T2DM. In our study, diabetes distress was associated with increased outpatient costs; diabetes with depressive symptoms may be linked to higher total medical expenditures. This may be due to the fact that diabetes distress did not reach the level of hospitalization and merely increased the use of outpatient services, thus only being associated with higher outpatient costs. People with T2DM with depressive symptoms would need to treat and cope with more severe psychological conditions, which led to higher total healthcare expenditures. Consistent with other studies, people with diabetes who had depression or distress had higher healthcare costs than those who did not (40, 50–52). A study using a nationally representative sample of the United States noted that among people with diabetes, the total expenditure on health management for adults with depression was 4.5 times that of adults without depression (41). The presence of comorbidity of depression and anxiety may expose more people with diabetes and their families to financial hardship (53). In addition, our research indicated that diabetes duration was one of the factors that affected drug costs. This may be because the longer the disease lasts, the greater the need for drugs to control the condition, and the corresponding increase in drug costs. The cost of diabetes drugs and equipment was a persistent obstacle to achieving glycemic goals (54). Co-occurring depressive symptoms may directly increase the financial burden of people with chronic conditions through psychotherapy, medical services, and the use of antidepressants (55).

Our findings raised the issue of whether necessary screening and care models needed to be developed for people with diabetes and comorbid psychological distress. Given the significant association of diabetes distress and depressive symptoms on the financial burden, specific population screening and monitoring of people with diabetes is critical. Treatment of depression in people with diabetes is both effective and cost-effective, improving overall outcomes (56). A study of a tiered collaborative care plan delivered by a nurse’s Depression Care Manager showed depression outcomes improved in the study intervention group compared with patients in the usual care group, with a 5-year average cost reduction of $3907 (57). Another study from the INDEPENDENT series indicated that multi-component collaborative care model can effectively improve combined psychological and metabolic outcomes of diabetic patients, likely by reducing depressive symptoms, enhancing self-management, and thereby improving the health outcomes (58–60). However, studies have shown that it is still common for adults with diabetes and depression not to receive antidepressants or psychotherapy for depression (61, 62). Therefore, diabetes distress should be monitored routinely (17). If diabetes distress is found, it should be acknowledged and dealt with (63). If necessary, patients should be referred for follow-up treatment (64). Focusing on the clinical predictors of distress and depression in people with diabetes and screening for people with diabetes appears to be a reasonable and feasible way to improve disease management. Adults with a history of depression need ongoing monitoring for relapse of depression in the context of usual care (65). A combination of mental and physical health care can improve medical outcomes for adults with a history of depression (63). A collaborative, person-centered approach to care has been shown to improve depression and medical outcomes (66).

The study has several limitations. First, this study is a cross-sectional study, which limits the inference of causal relationships in the results section, and it is also impossible to clarify whether there is a reverse causal inference between various variables. Future studies can further follow up on the population and adopt a cohort study design to further analyze the causal relationships between mental status, utilization of medical services, and medical service costs. Second, measures of depression symptoms or distress, and comorbidities were answered subjectively by the participants with interviewer assistance. The result may be underestimated, and individuals may choose better response options because of shame. Third, measures of healthcare utilization and expenditure were self-reported over a one-year recall period, which may be subject to recall bias. Fourth, we measured fasting blood glucose only once, without subsequent blood glucose monitoring, so it was impossible to describe changes in psychological conditions associated with changes in diabetes glycemic control, and whether psychiatric disorders lead to higher healthcare costs by exacerbating the development of diabetes. Fifth, the study was conducted in rural health clinics in southeastern China and may not be representative of populations in other regions. The results need to be generalized with caution. Sixth, our study did not consider the availability of health insurance, and future studies could add health insurance to study the impact on diabetes costs.

## 5 Conclusion

In conclusion, psychological distress (diabetes distress and depressive symptoms) are common in adults with T2DM. Psychological distress is associated with higher healthcare expenditures and more healthcare utilization. The results from our study suggest that diabetes distress is associated with higher outpatient costs and that diabetes with depressive symptoms is associated with higher total medical expenditures. This study reinforces the view that psychological screening and care interventions for adults with diabetes are necessary. Psychological guidance by a qualified mental health professional, preferably with diabetes expertise, is beneficial for improving the psychological status and health outcomes of adults with diabetes.

## Data Availability

The raw data supporting the conclusions of this article will be made available by the authors, without undue reservation.

## References

[1] GBD 2021 Diabetes Collaborators . Global, regional, and national burden of diabetes from 1990 to 2021, with projections of prevalence to 2050: a systematic analysis for the Global Burden of Disease Study 2021. Lancet. (2023) 402:203–34. doi: 10.1016/S0140-6736(23)01301-6, PMID: 37356446 PMC10364581

[2] Magliano DJ Boyko EJ IDF Diabetes Atlas 10th edition scientific committee . IDF DIABETES ATLAS. 10th ed. Brussels: International Diabetes Federation (2021). Available online at: http://www.ncbi.nlm.nih.gov/books/NBK581934/.

[3] Sun H Saeedi P Karuranga S Pinkepank M Ogurtsova K Duncan BB . IDF Diabetes Atlas: Global, regional and country-level diabetes prevalence estimates for 2021 and projections for 2045. Diabetes Res Clin Pract. (2022) 183:109119. doi: 10.1016/j.diabres.2021.109119, PMID: 34879977 PMC11057359

[4] Xue H Li P Luo Y Wu C Liu Y Qin X . Salidroside stimulates the Sirt1/PGC-1α axis and ameliorates diabetic nephropathy in mice. Phytomedicine. (2019) 54:240–7. doi: 10.1016/j.phymed.2018.10.031, PMID: 30668374

[5] Chen Y Hua Y Li X Arslan IM Zhang W Meng G . Distinct types of cell death and the implication in diabetic cardiomyopathy. Front Pharmacol. (2020) 11:42. doi: 10.3389/fphar.2020.00042, PMID: 32116717 PMC7018666

[6] Tu Y Song E Wang Z Ji N Zhu L Wang K . Melatonin attenuates oxidative stress and inflammation of Müller cells in diabetic retinopathy via activating the Sirt1 pathway. BioMed Pharmacother. (2021) 137:111274. doi: 10.1016/j.biopha.2021.111274, PMID: 33517190

[7] Zhang J Cai Z Yang M Tong L Zhang Y . Inhibition of tanshinone IIA on renin activity protected against osteoporosis in diabetic mice. Pharm Biol. (2020) 58:219–24. doi: 10.1080/13880209.2020.1738502, PMID: 32202179 PMC7144291

[8] Huang C-J Lin C-H Hsieh H-M Chang C-C Chu C-C Sun D-P . A longitudinal study of healthcare utilisation and expenditure in people with type 2 diabetes mellitus with and without major depressive disorder. Gen Hosp Psychiatry. (2019) 57:50–8. doi: 10.1016/j.genhosppsych.2018.09.007, PMID: 30908962

[9] Owens-Gary MD Zhang X Jawanda S Bullard KM Allweiss P Smith BD . The importance of addressing depression and diabetes distress in adults with type 2 diabetes. J Gen Intern Med. (2019) 34:320–4. doi: 10.1007/s11606-018-4705-2, PMID: 30350030 PMC6374277

[10] Zurita-Cruz JN Manuel-Apolinar L Arellano-Flores ML Gutierrez-Gonzalez A Najera-Ahumada AG Cisneros-González N . Health and quality of life outcomes impairment of quality of life in type 2 diabetes mellitus: a cross-sectional study. Health Qual Life Outcomes. (2018) 16:94. doi: 10.1186/s12955-018-0906-y, PMID: 29764429 PMC5952418

[11] Farooqi A Gillies C Sathanapally H Abner S Seidu S Davies MJ . A systematic review and meta-analysis to compare the prevalence of depression between people with and without Type 1 and Type 2 diabetes. Primary Care Diabetes. (2022) 16:1–10. doi: 10.1016/j.pcd.2021.11.001, PMID: 34810141

[12] Liu X Dong C Jiang H Zhong D Li Y Zhang H . Prevalence and risk factors of depression in Chinese patients with type 2 diabetes mellitus: a protocol of systematic review and meta-analysis. Syst Rev. (2021) 10:302. doi: 10.1186/s13643-021-01855-7, PMID: 34823606 PMC8620640

[13] Perrin NE Davies MJ Robertson N Snoek FJ Khunti K . The prevalence of diabetes-specific emotional distress in people with Type 2 diabetes: a systematic review and meta-analysis. Diabetes Med. (2017) 34:1508–20. doi: 10.1111/dme.13448, PMID: 28799294

[14] Wong EM Afshar R Qian H Zhang M Elliott TG Tang TS . Diabetes distress, depression and glycemic control in a canadian-based specialty care setting. Can J Diabetes. (2017) 41:362–5. doi: 10.1016/j.jcjd.2016.11.006, PMID: 28462795

[15] Fisher L Skaff MM Mullan JT Arean P Glasgow R Masharani U . A longitudinal study of affective and anxiety disorders, depressive affect and diabetes distress in adults with Type 2 diabetes. Diabetes Med. (2008) 25:1096–101. doi: 10.1111/j.1464-5491.2008.02533.x, PMID: 19183314 PMC2635496

[16] Kamrul-Hasan ABM Hannan MA Asaduzzaman M Rahman MM Alam MS Amin MN . Prevalence and predictors of diabetes distress among adults with type 2 diabetes mellitus: a facility-based cross-sectional study of Bangladesh. BMC Endocr Disord. (2022) 22:28. doi: 10.1186/s12902-022-00938-3, PMID: 35065623 PMC8783990

[17] Snoek FJ Bremmer MA Hermanns N . Constructs of depression and distress in diabetes: time for an appraisal. Lancet Diabetes Endocrinol. (2015) 3:450–60. doi: 10.1016/S2213-8587(15)00135-7, PMID: 25995123

[18] Deng C Lu C Wang K Chang M Shen Y Yang X . Celecoxib ameliorates diabetic sarcopenia by inhibiting inflammation, stress response, mitochondrial dysfunction, and subsequent activation of the protein degradation systems. Front Pharmacol. (2024) 15:1344276. doi: 10.3389/fphar.2024.1344276, PMID: 38313305 PMC10834620

[19] Chen D Huang C Chen Z . A review for the pharmacological effect of lycopene in central nervous system disorders. BioMed Pharmacother. (2019) 111:791–801. doi: 10.1016/j.biopha.2018.12.151, PMID: 30616078

[20] Zhang C Yu H Yang H Liu B . Activation of PI3K/PKB/GSK-3β signaling by sciadopitysin protects cardiomyocytes against high glucose-induced oxidative stress and apoptosis. J Biochem Mol Toxicol. (2021) 35:e22887. doi: 10.1002/jbt.22887, PMID: 34392578

[21] Lu J Ma Y Wu J Huang H Wang X Chen Z . A review for the neuroprotective effects of andrographolide in the central nervous system. BioMed Pharmacother. (2019) 117:109078. doi: 10.1016/j.biopha.2019.109078, PMID: 31181444

[22] Li S Yang D Zhou X Chen L Liu L Lin R . Neurological and metabolic related pathophysiologies and treatment of comorbid diabetes with depression. CNS Neurosci Ther. (2024) 30:e14497. doi: 10.1111/cns.14497, PMID: 37927197 PMC11017426

[23] Boden MT . Prevalence of mental disorders and related functioning and treatment engagement among people with diabetes. J Psychosom Res. (2018) 106:62–9. doi: 10.1016/j.jpsychores.2018.01.001, PMID: 29455901

[24] Lin K Park C Li M Wang X Li X Li W . Effects of depression, diabetes distress, diabetes self-efficacy, and diabetes self-management on glycemic control among Chinese population with type 2 diabetes mellitus. Diabetes Res Clin Pract. (2017) 131:179–86. doi: 10.1016/j.diabres.2017.03.013, PMID: 28756132

[25] Gao Y Xiao J Han Y Ji J Jin H Mawen DG . Self-efficacy mediates the associations of diabetes distress and depressive symptoms with type 2 diabetes management and glycemic control. Gen Hosp Psychiatry. (2022) 78:87–95. doi: 10.1016/j.genhosppsych.2022.06.003, PMID: 35932599

[26] Hutter N Schnurr A Baumeister H . Healthcare costs in patients with diabetes mellitus and comorbid mental disorders—a systematic review. Diabetologia. (2010) 53:2470–9. doi: 10.1007/s00125-010-1873-y, PMID: 20700575

[27] Su C-H Chiu H-C Hsieh H-M Yen J-Y Lee M-H Li C-Y . Healthcare utilization and expenditures for persons with diabetes comorbid with mental illnesses. Psychiatr Q. (2016) 87:545–57. doi: 10.1007/s11126-015-9408-9, PMID: 26646577

[28] Shrestha SS Zhang P Li R Thompson TJ Chapman DP Barker L . Medical expenditures associated with major depressive disorder among privately insured working-age adults with diagnosed diabetes in the United States, 2008. Diabetes Res Clin Pract. (2013) 100:102–10. doi: 10.1016/j.diabres.2013.02.002, PMID: 23490596 PMC5304910

[29] Sporinova B Manns B Tonelli M Hemmelgarn B MacMaster F Mitchell N . Association of mental health disorders with health care utilization and costs among adults with chronic disease. JAMA Netw Open. (2019) 2:e199910. doi: 10.1001/jamanetworkopen.2019.9910, PMID: 31441939 PMC6714022

[30] Hutter N Scheidt-Nave C Baumeister H . Health care utilisation and quality of life in individuals with diabetes and comorbid mental disorders. Gen Hosp Psychiatry. (2009) 31:33–5. doi: 10.1016/j.genhosppsych.2008.09.008, PMID: 19134508

[31] Fan Y He D Chen L Ge C . Association between the depressive symptom trajectories and all-cause mortality in Chinese middle-aged and elderly adults. Sci Rep. (2025) 15:879. doi: 10.1038/s41598-025-85177-x, PMID: 39762339 PMC11704258

[32] Malhi GS Mann JJ . Depression. Lancet. (2018) 392:2299–312. doi: 10.1016/S0140-6736(18)31948-2, PMID: 30396512

[33] Polonsky WH Fisher L Earles J Dudl RJ Lees J Mullan J . Assessing psychosocial distress in diabetes: development of the diabetes distress scale. Diabetes Care. (2005) 28:626–31. doi: 10.2337/diacare.28.3.626, PMID: 15735199

[34] Fisher L Hessler DM Polonsky WH Mullan J . When is diabetes distress clinically meaningful?: establishing cut points for the diabetes distress scale. Diabetes Care. (2012) 35:259–64. doi: 10.2337/dc11-1572, PMID: 22228744 PMC3263871

[35] Ting RZW Nan H Yu MWM Kong APS Ma RCW Wong RYM . Diabetes-related distress and physical and psychological health in chinese type 2 diabetic patients. Diabetes Care. (2011) 34:1094–6. doi: 10.2337/dc10-1612, PMID: 21398526 PMC3114496

[36] Andresen EM Malmgren JA Carter WB Patrick DL . Screening for depression in well older adults: evaluation of a short form of the CES-D (Center for Epidemiologic Studies Depression Scale). Am J Prev Med. (1994) 10:77–84. doi: 10.1016/S0749-3797(18)30622-6, PMID: 8037935

[37] Lei X Sun X Strauss J Zhang P Zhao Y . Depressive symptoms and SES among the mid-aged and elderly in China: evidence from the China Health and Retirement Longitudinal Study national baseline. Soc Sci Med. (1982) 2014) 120:224–32. doi: 10.1016/j.socscimed.2014.09.028, PMID: 25261616 PMC4337774

[38] Mansfield ER Helms BP . Detecting multicollinearity. Am Statistician. (1982) 36:158–60. doi: 10.2307/2683167

[39] Egede LE Ellis C . Diabetes and depression: Global perspectives. Diabetes Res Clin Pract. (2010) 87:302–12. doi: 10.1016/j.diabres.2010.01.024, PMID: 20181405

[40] Egede LE Dismuke CE . Serious psychological distress and diabetes: a review of the literature. Curr Psychiatry Rep. (2012) 14:15–22. doi: 10.1007/s11920-011-0240-0, PMID: 22002804

[41] Egede LE Zheng D Simpson K . Comorbid depression is associated with increased health care use and expenditures in individuals with diabetes. Diabetes Care. (2002) 25:464–70. doi: 10.2337/diacare.25.3.464, PMID: 11874931

[42] Fisher L Skaff MM Mullan JT Arean P Mohr D Masharani U . Clinical depression versus distress among patients with type 2 diabetes: not just a question of semantics. Diabetes Care. (2007) 30:542–8. doi: 10.2337/dc06-1614, PMID: 17327318

[43] Tareen RS Tareen K . Psychosocial aspects of diabetes management: dilemma of diabetes distress. Transl Pediatr. (2017) 6:383–96. doi: 10.21037/tp.2017.10.04, PMID: 29184819 PMC5682378

[44] Skinner TC Joensen L Parkin T . Twenty-five years of diabetes distress research. Diabetes Med: J Br Diabetes Assoc. (2020) 37:393–400. doi: 10.1111/dme.14157, PMID: 31638279

[45] Larsen A Pintye J Odhiambo B Mwongeli N Marwa MM Watoyi S . Comparing depression screening tools (CESD-10, EPDS, PHQ-9, and PHQ-2) for diagnostic performance and epidemiologic associations among postpartum Kenyan women: Implications for research and practice. J Affect Disord. (2023) 324:637–44. doi: 10.1016/j.jad.2022.12.101, PMID: 36586607 PMC9990497

[46] Bergenfeld I Kaslow NJ Yount KM Cheong YF Johnson ER Clark CJ . Measurement invariance of the center for epidemiologic studies scale-depression within and across six diverse intervention trials. Psychol Assess. (2023) 35:805–20. doi: 10.1037/pas0001262, PMID: 37616094 PMC10662958

[47] Cooper Z Johnson L Ali MK Patel SA Poongothai S Mohan V . Factors influencing diabetes treatment satisfaction in the integrating depression and diabetes treatment randomized clinical trial: a multilevel model analysis. Diabetes Med. (2024) 41:e15412. doi: 10.1111/dme.15412, PMID: 39039715 PMC11560630

[48] Nichols L Barton PL Glazner J McCollum M . Diabetes, minor depression and health care utilization and expenditures: a retrospective database study. Cost Effectiveness Resource Allocation. (2007) 5:4. doi: 10.1186/1478-7547-5-4, PMID: 17442110 PMC1863422

[49] ElSayed NA Aleppo G Aroda VR Bannuru RR Brown FM Bruemmer D . 2. Classification and diagnosis of diabetes: standards of care in diabetes—2023. Diabetes Care. (2022) 46:S19–40. doi: 10.2337/dc23-S002, PMID: 36507649 PMC9810477

[50] Al Qusaibi B Mosli H Kattan W Fadel H Alariefy A Almalki B . Depression among patients with type 2 diabetes mellitus at king abdulaziz university hospital (KAUH): A cross-sectional study. Cureus. (2022) 14:e25990. doi: 10.7759/cureus.25990, PMID: 35855231 PMC9286297

[51] Akena D Kadama P Ashaba S Akello C Kwesiga B Rejani L . The association between depression, quality of life, and the health care expenditure of patients with diabetes mellitus in Uganda. J Affect Disord. (2015) 174:7–12. doi: 10.1016/j.jad.2014.11.019, PMID: 25479048 PMC4549461

[52] Le TK Able SL Lage MJ . Resource use among patients with diabetes, diabetic neuropathy, or diabetes with depression. Cost Effectiveness Resource Allocation. (2006) 4:18. doi: 10.1186/1478-7547-4-18, PMID: 17059602 PMC1629026

[53] Wallace K Zhao X Misra R Sambamoorthi U . The humanistic and economic burden associated with anxiety and depression among adults with comorbid diabetes and hypertension. J Diabetes Res. (2018) 2018:4842520. doi: 10.1155/2018/4842520, PMID: 30474044 PMC6220385

[54] ElSayed NA Aleppo G Aroda VR Bannuru RR Brown FM Bruemmer D . 1. Improving care and promoting health in populations: standards of care in diabetes—2023. Diabetes Care. (2022) 46:S10–8. doi: 10.2337/dc23-S001, PMID: 36507639 PMC9810463

[55] Wu Y Jin S Guo J Zhu Y Chen L Huang Y . The economic burden associated with depressive symptoms among middle-aged and elderly people with chronic diseases in China. Int J Environ Res Public Health. (2022) 19:12958. doi: 10.3390/ijerph191912958, PMID: 36232268 PMC9566659

[56] Simon GE Katon WJ Lin EHB Rutter C Manning WG Von Korff M . Cost-effectiveness of systematic depression treatment among people with diabetes mellitus. Arch Gen Psychiatry. (2007) 64:65–72. doi: 10.1001/archpsyc.64.1.65, PMID: 17199056

[57] Katon WJ Russo JE Von Korff M Lin EHB Ludman E Ciechanowski PS . Long-term effects on medical costs of improving depression outcomes in patients with depression and diabetes. Diabetes Care. (2008) 31:1155–1159. doi: 10.2337/dc08-0032, PMID: 18332158 PMC3810023

[58] Ali MK Chwastiak L Poongothai S Emmert-Fees KMF Patel SA Anjana RM . Effect of a collaborative care model on depressive symptoms and glycated hemoglobin, blood pressure, and serum cholesterol among patients with depression and diabetes in India: the INDEPENDENT randomized clinical trial. JAMA. (2020) 324:651–62. doi: 10.1001/jama.2020.11747, PMID: 32809002 PMC7435347

[59] Cooper ZW Johnson LCM Patel SA Aravind SR Tandon N Anjana RM . The influence of depression, positive health behaviors, and weight status on glycated hemoglobin: a sequential mediation analysis of the INDEPENDENT trial. J Gen Intern Med. (2025) 40:3715–22. doi: 10.1007/s11606-025-09810-1, PMID: 40804563 PMC12612419

[60] Hassan S Liu S Johnson LCM Patel SA Emmert-Fees KMF Suvada K . Association of collaborative care intervention features with depression and metabolic outcomes in the INDEPENDENT study: A mixed methods study. Primary Care Diabetes. (2024) 18:319–26. doi: 10.1016/j.pcd.2024.02.001, PMID: 38360505 PMC11127790

[61] Nicolucci A Kovacs Burns K Holt RIG Comaschi M Hermanns N Ishii H . Diabetes Attitudes, Wishes and Needs second study (DAWN2TM): Cross-national benchmarking of diabetes-related psychosocial outcomes for people with diabetes. Diabetic Med. (2013) 30:767–77. doi: 10.1111/dme.12245, PMID: 23711019

[62] Alenzi EO Sambamoorthi U . Depression treatment and health-related quality of life among adults with diabetes and depression. Qual Life Res. (2016) 25:1517–25. doi: 10.1007/s11136-015-1189-y, PMID: 26590839 PMC4888865

[63] ElSayed NA Aleppo G Aroda VR Bannuru RR Brown FM Bruemmer D . 5. Facilitating positive health behaviors and well-being to improve health outcomes: standards of care in diabetes—2023. Diabetes Care. (2022) 46:S68–96. doi: 10.2337/dc23-S005, PMID: 36507648 PMC9810478

[64] McMorrow R Hunter B Hendrieckx C Kwasnicka D Speight J Cussen L . Effect of routinely assessing and addressing depression and diabetes distress on clinical outcomes among adults with type 2 diabetes: a systematic review. BMJ Open. (2022) 12:e054650. doi: 10.1136/bmjopen-2021-054650, PMID: 35613752 PMC9134162

[65] Lustman PJ Griffith LS Clouse RE . Depression in adults with diabetes. Results of 5-yr follow-up study. Diabetes Care. (1988) 11:605–12. doi: 10.2337/diacare.11.8.605, PMID: 3219966

[66] Katon WJ Von Korff M Lin EHB Simon G Ludman E Russo J . The Pathways Study: a randomized trial of collaborative care in patients with diabetes and depression. Arch Gen Psychiatry. (2004) 61:1042–9. doi: 10.1001/archpsyc.61.10.1042, PMID: 15466678

