# Supplementary material for: Healthcare utilization and expenditures among adults with type 2 diabetes mellitus and comorbid psychological distress
Source: Front Endocrinol (Lausanne). 2026 Jan 29;17:1702996. doi: 10.3389/fendo.2026.1702996 (PMC12893985; doi:10.3389/fendo.2026.1702996)
Supplement: Supplementary file 1 [file DataSheet1.docx]

**Additional Questionnaire: Basic Public Health Service Cognition questionnaire**

**for chronic patients**

Institution: Institution code: Questionnaire number:

Investigator: Quality controller:

| **serial number** | **content** | answer |
| --- | --- | --- |
| A1 | Name: |  |
| A2 | ID number: |  |
| A3 | Date of birth: A3a Year A3b moon |  |
| A4 | Gender: 1. Male 2. Female |  |
| A5 | Education: 1. Below primary 2. Primary 3. Junior High 4. High School or technical secondary 5. College 6. Bachelor's degree or above |  |
| A6 | Current marital status: 1. Unmarried 2. Married and Living Together 3. Married but separated 4. Divorced 5. Widowed 6. Others |  |
| A7 | Your current employment status:  1.Cadres (including doctors, teachers and other institutions)  2. Self-employed activities (including farmers and small business owners)  3. Temporary work (including paid family activities)  4. Unpaid domestic helpers  5 workers, formal jobs employed by units or individuals (including enterprises and institutions, collective enterprises/farms or private enterprises)  6. Leisure, doing nothing  7. Others |  |
| A8 | In the past year, the permanent population in your family (those who have lived at home for more than 6 months) A8a People,  A8b Among them, the elderly over 65 years old people. |  |
| A9 | In the past year, the total income of all your families was about ten thousand |  |

(2) Past medical history of diabetes and self-assessment of health

| B1 | When was the first time you were diagnosed with diabetes by a doctor? B1a. Year B1b. moon |  |
| --- | --- | --- |
| B2 | Do your immediate family members (grandparents, parents, brothers and sisters) have diabetes? 1.Yes 2. no |  |
| B3 | Which of the following chronic diseases have you been diagnosed by doctors (multiple choices are allowed)  1. Not diagnosed with any chronic diseases. 2. Cardiovascular diseases (such as myocardial infarction, coronary heart disease, congestive heart failure and others)  3. Cerebrovascular diseases (cerebral hemorrhage, cerebral arteriosclerosis, stroke) 4. Hypertension  5. Chronic lung diseases (such as chronic bronchitis, emphysema and asthma) 6. Malignant tumor  7. Other chronic diseases (please note) |  |
| B4 | What measures do you take to control diabetes? (Multiple choices are allowed)  1. Take the medicine according to the doctor's advice 2. inject insulin 3. control your diet  4. moderate exercise 5. No measures have been taken |  |
| B5 | Generally speaking, how do you rate your health in the past month?  1. Very good, 2. Good, 3. Fair, 4. Poor, 5. Very poor. |  |
|  | EQ-5D Quality of Life (Health Status Today) |  |
| B6 | Action  1. You can walk around without any difficulty. 2. It's a little inconvenient 3. Can't get out of bed and move. |  |
| B7 | Take care of yourself (in washing face, brushing teeth, bathing or dressing)  1.There is no difficulty 2. There are some difficulties 3. I can't do it by myself. |  |
| B8 | Daily activities (such as work, housework, family or leisure activities)  1. There are no difficulties. 2. There are some difficulties. 3. Unable to proceed |  |
| B9 | Pain/discomfort  1. There is nothing 2. Moderate 3. Extreme pain or discomfort |  |
| B10 | Anxiety (such as nervousness, anxiety, anxiety, etc.)/depression (such as lack of interest, lack of fun, lack of energy, etc.).  1. Don't feel anxious or depressed 2. Moderate anxiety or depression 3. Extreme anxiety or depression |  |
| B11 | In order to help you reflect the quality of your health, we drew a scale (a bit like a thermometer). On this scale, 100 represents the best condition in your mind and 0 represents the worst condition in your mind. Please mark your health today on the scale below.  0 10 20 30 40 50 60 70 80 90 100 |  |

(3) Health-related behaviors and drug compliance

| C1 | Have you smoked in the past three months?  1. Never suck (skip C2) 2. Occasionally (skip C2) 3. Often. 4. Smoking every day. 5. Quitting smoking (skip C2) |  |
| --- | --- | --- |
| C1a | If you smoke, average daily smoking . C1b .you have been smoking for years |  |
|  | **Leisure exercise situation** |  |
| C2 | Have you ever done strenuous exercise or leisure activities that **lasted at least 10 minutes and caused a significant increase in breathing and heartbeat?** Such as long-distance running and playing ball, fast run, etc. 1. yes 2. No (skip C5) |  |
| C3 | Usually within a week, you have days to do the above strenuous exercise or leisure activities every day? |  |
| C4 | **How long do you spend doing the above strenuous sports or leisure activities in a day?**  **hour** **minute** |  |
| C5 | **Have you ever done moderate-intensity exercise or leisure activities that lasted at least 10 minutes and caused a significant increase in breathing and heartbeat?** Dance, brisk walking, swimming, playing volleyball, etc. 1. yes 2. No (skip C8) |  |
| C6 | Usually within a week, you have days to do the above moderate-intensity sports or leisure activities? |  |
| C7 | How long do you spend doing the above moderate-intensity sports or leisure activities in a day?  hour minute |  |
| C8 | How much time do you spend sitting, leaning or lying down in a day? (including sitting, working, studying, reading, watching TV, using the computer, doing manual work, resting and other static behaviors, but not including sleeping time) hour minute |  |
| C9 | How long do you sleep in a day? evening hours ; at noon and other times hours |  |
| C10 | How do you feel about your sleep quality? 1. Very good 2. Good 3. Fair 4. Poor 5. Very poor |  |
|  | **Drug compliance (Morisky compliance behavior scale)** |  |
| C11 | Have you ever forgotten to use diabetes drugs? 1. Yes 2. No. |  |
| C12 | In the past two weeks, have you forgotten to take medicine for one or more days? 1. Yes 2. No. |  |
| C13 | When you feel the symptoms get worse or other symptoms appear, do you reduce the dosage or stop taking the medicine without telling the doctor? 1. Yes 2. No. |  |
| C14 | When you travel or leave home for a long time, have you ever forgotten to bring diabetes drugs? 1. Yes 2. No. |  |
| C15 | Did you take diabetes drugs yesterday? 1. Yes 2. No. |  |
| C16 | When you feel that your symptoms have improved or disappeared, have you stopped taking medicine?  1. Yes 2. No. |  |
| C17 | Do you find it difficult to stick to the treatment plan of controlling blood sugar? 1. Yes 2. No. |  |
| C18 | Do you think it is difficult to remember to take diabetes drugs on time and in quantity?  1. Never 2. Occasionally 3. Sometimes 4. Often 5. All the time. |  |
|  | **Doctor trust scale** |  |
| C19 | Would you recommend your doctor to your friends: 1. Very willing 2. More willing 3. Unwilling 4. Less willing 5. Very unwilling |  |
| C20 | You had an argument with the doctor:1. It keeps happening. 2. It often happens. 3. Sometimes it happens. 4. It rarely happens. 5 Never happen. |  |
| C21 | You will find another doctor to diagnose you: 1. Sure. 2.It may be. 3. No. 4. Not really. 5. Definitely not. |  |
| C22 | You take the medicine according to the doctor's instructions. 1. Exactly. 2. Sometimes. 3. Occasionally. 4. Very few. 5. Not at all. |  |
| C23 | You want to change to another doctor to serve you: 1. I hope very much; 2. I hope rather. 3. I don't want to. 4. Don't quite agree. 5. Strongly disagree. |  |

**(4) the awareness and satisfaction of basic public health services**

| D1 | You know that the government provides residents with basic public health services free of charge (such as those provided by nearby community hospitals, town health centers and village clinics, physical examination for the elderly, health services for hypertension and diabetes, health knowledge lectures)?  1. know 2. I don't know |  |
| --- | --- | --- |
| D2 | Do you know what public health services provided by the state include? (multiple choice questions)  1. Vaccination (vaccination for school-age children)  2. Children's health care (children should be weighed, measured and checked for child care)   1. Health management of hypertension/diabetes (free measurement of blood pressure, blood sugar and telephone, face-to-face health guidance) 2. Health education (community hospitals attend health knowledge lectures and community doctors provide health consultation) 3. Chinese medicine health care (providing Chinese medicine physique assessment and Chinese medicine health guidance for the elderly) 4. Maternal health care (prenatal physical examination, postpartum family visit) 5. Establish health records of residents (fill in the health record form after community doctors register personal health information) 6. Health care for the elderly (provide free physical examination and health guidance for the elderly over 65 years old every year) 7. Infectious disease management 10. Health management of patients with severe mental illness   11. Health management services for tuberculosis patients  12. Health and family planning supervision and co-management 13. Provide contraceptives free of charge  14. Health literacy promotion actions |  |
| D3 | Which of the above services have you and your family enjoyed? (multiple choice questions) |  |
| D4 | Generally speaking, are you and your family satisfied with the basic public health services you have enjoyed?  1. Very dissatisfied 2. Not very satisfied 3. Fair 4. Relatively satisfied 5. Very satisfied |  |

**(5) Health knowledge literacy**

| E1 | What do you think is the normal blood pressure value (high pressure/low pressure) of adults, and it is diagnosed as hypertension?  1.150/100 mmHg 2. 140/90 mmHg 3. 120 /80 mmHg 4. I don't know |  |  |
| --- | --- | --- | --- |
| E2 | Smoking and passive smoking can cause many diseases such as cancer, cardiovascular and respiratory system.  1. Right 2. Wrong 3. I don't know |  |  |
| E3 | What is the correct dietary method to prevent obesity?  1. Eat more fried food 2. Eat more foods with high fat content 3. Eat lightly and control your food intake  4. I don't know |  |  |
| E4 | Which of the following behaviors can prevent AIDS? 1. Prevent mosquito bites 2. share razors 3. share tableware 4. I don't know |  |  |
| E5 | What do you think is the most effective way to prevent hepatitis B? 1. Pay attention to washing hands before meals 2. Eat more animal livers 3. inject hepatitis b vaccine 4. I don't know |  |  |
| E6 | What should the average adult eat? 1. Mainly vegetables/fruits. 2. Mainly meat, eggs and milk. 3. Mainly cereals. 4. I don't know. |  |  |
| E7 | What do you think is true of the following statement:  1. Raw meat should use different chopping boards from cooked meat. 2. After cutting raw meat, you can cut cooked meat with water. 3. The chopping board for cutting raw meat can be washed with water to cut cooked meat. 4. I don't know. |  |  |
| E8 | Regarding the understanding of mental health, what do you think is correct:  1. Having a psychological problem means having a mental illness. 2. Ask for help when you have psychological problems. 3. If you have psychological problems, just keep them in your mind, without asking for help. 4. I don't know. |  | |
| E9 | Do you smoke more than one cigarette a day for more than half a year? 1. Yes 2. No |  | |
| E10 | Do you have the habit of washing your hands before and after meals?  1. Never 2. occasionally 3. Often 4. Always |  | |
| E11 | When do you usually brush your teeth? 1.brush in the morning 2.brush at night 3. Brush in the morning and evening 4. Rarely brush |  | |
| E12 | How many hours do you sleep every day?  1. Less than 6 hours 2. 6~7 hours 3. 7~8 hours 4. 8~9 hours 5. More than 9 hours |  | |
| E13 | Your habit of doing physical exercises (such as running, playing ball, tai chi, doing exercises, dancing, etc., lasting more than half an hour at a time) is:  1. little exercise 2. 1~3 times a month 3. Once a week 4. 2~3 times a week 5. 4~7 times a week |  | |
| E14 | Without a doctor's prescription, would you go to the drugstore to buy antibiotics? 1. Yes 2. no |  | |
| E15 | Do you have a physical examination once a year? 1. Yes 2. No |  | |
| E16 | Did you feel stressed in the past year? 1. Never 2. occasionally 3. Often 4. Always |  | |
| E17 | When you need emergency medical assistance, you should call? 1.120 2.119 3.122 4. I don't know |  | |

**(6) Self-management Behavior Scale for Diabetic Patients**

| serial number | How about your diet, exercise and self-examination in the past 7 days? | 0 | 1 | 2 | 3 | 4 | 5 | 6 | 7 | answer |
| --- | --- | --- | --- | --- | --- | --- | --- | --- | --- | --- |
| F1 | How many days do you have to eat according to a healthy diet plan? | 0 | 1 | 2 | 3 | 4 | 5 | 6 | 7 |  |
| F2 | In the past month, how many days per week did you reasonably arrange your diet according to the dietary requirements of diabetes? | 0 | 1 | 2 | 3 | 4 | 5 | 6 | 7 |  |
| F3 | How many days do you eat more than 5 servings of vegetables and fruits a day, such as eating 3 dishes of vegetables and water; 2 fruits (one fruit is about one orange; 200 grams of vegetables, about 1 dish) | 0 | 1 | 2 | 3 | 4 | 5 | 6 | 7 |  |
| F4 | How many days do you eat greasy food (such as fried food, fat meat, chicken skin, etc.) | 0 | 1 | 2 | 3 | 4 | 5 | 6 | 7 |  |
| F5 | How many days have you done activities for more than 30 minutes (referring to continuous physical activity for more than 30 minutes, Including: walking, doing housework) | 0 | 1 | 2 | 3 | 4 | 5 | 6 | 7 |  |
| F6 | Apart from work and housework, how many days do you have to do sports (such as jogging, square dancing, cycling, mountain climbing, Tai Ji Chuan, playing ball games, swimming, etc.)? | 0 | 1 | 2 | 3 | 4 | 5 | 6 | 7 |  |
| F7 | How many days do you measure your blood sugar at home (or with the help of your family)? | 0 | 1 | 2 | 3 | 4 | 5 | 6 | 7 |  |
| F8 | How many days do you have to measure your blood sugar at home on time (or with the help of your family) according to the standard times of blood sugar measurement instructed by your doctor (for example, twice a day)? | 0 | 1 | 2 | 3 | 4 | 5 | 6 | 7 |  |
| F9 | How many days do you have to check your feet (including toes, soles and soles)? | 0 | 1 | 2 | 3 | 4 | 5 | 6 | 7 |  |
| F10 | How many days have you checked the situation inside your shoes before you put them on (for example, are there any small stones in your shoes? Whether it is flat, damaged or wet, etc.) | 0 | 1 | 2 | 3 | 4 | 5 | 6 | 7 |  |
| F11 | How many days do you take hypoglycemic drugs or inject insulin regularly and quantitatively according to the doctor's instructions? | 0 | 1 | 2 | 3 | 4 | 5 | 6 | 7 |  |

**(7) Behavioral changes of diabetes mellitus**

| serial number | For each of the following questions, please tick the option that truly reflects your confidence in completing these daily tasks now. How confident are you that you can ...... | 1.  Full confidence | 2. Most confidence. | 3. Sometimes have confidence | 4.  Most have no confidence | 5.  No confidence at all | answer |
| --- | --- | --- | --- | --- | --- | --- | --- |
| K1 | Take medicine regularly according to the doctor's advice for a long time. |  |  |  |  |  |  |
| K2 | Achieve a low-salt diet. |  |  |  |  |  |  |
| K3 | Resist the temptation of delicious food and reduce the intake of high-fat food. |  |  |  |  |  |  |
| K4 | Exercise 4-5 days a week and exercise for more than 30 minutes every day. |  |  |  |  |  |  |
| K5 | Quit smoking successfully. |  |  |  |  |  |  |
| K6 | Limit the amount of alcohol consumed. |  |  |  |  |  |  |
| K7 | Don't let your discomfort or pain caused by illness affect what you want to do. |  |  |  |  |  |  |
| K8 | Don't let the depression caused by illness affect what you want to do. |  |  |  |  |  |  |

(8) Personal/family medical expenses and economic situation (in the past year)

| G1 | Have you been hospitalized for any length of time in the past year? 1. Yes 2. No (skip to G6) |  |
| --- | --- | --- |
| G2 | G2a hospitalization Times, G2b cumulative hospitalization days: Days |  |
| G4 | How much did the hospitalization medical expenses cost (including registration, examination, medicine purchase and treatment, including medical insurance reimbursement and out-of-pocket expenses)?  G4a Yuan; G4b Among them, the total cost of simple drugs (including medical insurance reimbursement and out-of-pocket expenses). Yuan |  |
| G5 | How much did you spend on other expenses besides medical expenses (such as accommodation, transportation, escort, etc.) Yuan. |  |
| G6 | Have you had a doctor visit or other healthcare professional in the past year? Have you had any outpatient visits or emergency department visits in the past year, including preventive and acute care? 1. Yes 2. No (skip to G9) G7. Number of outpatient visits times |  |
| G8 | How much did the outpatient medical expenses cost (including registration, examination, medicine purchase, treatment and other expenses, including medical insurance reimbursement and out-of-pocket expenses)?  G8a Yuan; How much is the total cost of G8b pure medicine (including medical insurance reimbursement and out-of-pocket expenses)? Yuan |  |
| G9 | How much did you spend on going to the drugstore in the past year (including medicines, instruments, test paper and health products)? Yuan. |  |
| G10 | What is the average monthly living expenses of your family in the past year? Yuan/month |  |
| G11 | The average monthly total expenditure on hypertension (including physical examination, drug purchase, hospitalization, etc.) is Yuan/month |  |

**(9)Mental health survey and evaluation form**

| serial number | Did you have the following situations in the past week: | 1. rarely or none.  (< 1 day) | 2. Not too much  (1-2 days) | 3. Sometimes  (3-4 days) | 4. Most  (5-7 days) | answer |
| --- | --- | --- | --- | --- | --- | --- |
| H1 | I'm troubled by little things. |  |  |  |  |  |
| H2 | It's hard for me to concentrate when I'm doing something. |  |  |  |  |  |
| H3 | I feel depressed. |  |  |  |  |  |
| H4 | I find it hard to do anything. |  |  |  |  |  |
| H5 | I am full of hope for the future. |  |  |  |  |  |
| H6 | I feel scared. |  |  |  |  |  |
| H7 | I don't sleep well. |  |  |  |  |  |
| H8 | I am very happy. |  |  |  |  |  |
| H9 | I feel lonely. |  |  |  |  |  |
| H10 | I don't think I can go on with my life. |  |  |  |  |  |

**(10) Mental distress scale**

|  | **In the past 7 days, for each of the following questions, please choose the option that truly reflects your current situation. Do you have any problems with the following options?** | 1.  None | 2.  mild | 3.  moderate | 4.  Slightly severe | 5.  Relatively severe | 6.  severe | answer | |  |
| --- | --- | --- | --- | --- | --- | --- | --- | --- | --- | --- |
| J1 | Your health management doctor lacks knowledge about diabetes and nursing. |  |  |  |  |  |  |  | |  |
| J2 | You feel that diabetes consumes a lot of energy and physical strength every day. |  |  |  |  |  |  |  | |  |
| J3 | You are not confident in your daily ability to deal with diabetes. |  |  |  |  |  |  |  | |  |
| J4 | Whenever you think of life with diabetes, you feel angry, afraid and or depressed. |  |  |  |  |  |  |  | |  |
| J5 | Your doctor didn't give clear instructions on how to manage diabetes. |  |  |  |  |  |  |  | |  |
| J6 | It is impossible to measure blood sugar frequently. |  |  |  |  |  |  |  | |  |
| J7 | No matter what you do, your life will end with long-term serious complications. |  |  |  |  |  |  |  | |  |
| J8 | Because of some trivial things about diabetes, you often feel that everything is not going well. |  |  |  |  |  |  |  | |  |
| J9 | Friends or family members are not supportive enough, and your efforts in self-management of diabetes.  (For example, planned activities conflict with my life schedule and encourage me to eat high-sugar food) |  |  |  |  |  |  |  | |  |
| J10 | Diabetes controls your life. |  |  |  |  |  |  |  | |  |
| J11 | Your doctor didn't take your concerns about diabetes seriously. |  |  |  |  |  |  |  | |  |
| J12 | Not strictly implementing the dietary guidance program for diabetes. |  |  |  |  |  |  |  | |  |
| J13 | The life of diabetics is very difficult, and your friends or family can't understand it. |  |  |  |  |  |  |  | |  |
| J14 | Because of diabetes, you often have some needs in your life, which makes you feel at a loss. |  |  |  |  |  |  |  | |  |
| J15 | You don't have a doctor who pays close attention to your diabetes very regularly. |  |  |  |  |  |  |  | |  |
| J16 | You are not active enough to insist on self-management of diabetes. |  |  |  |  |  |  |  | |  |
| J17 | Your friends or family members can't give you the emotional support you want. |  |  |  |  |  |  |  | |  |
| Physical examination index | M1 height centimetre M2 weight kilogram M3 waist circumference centimetre | | | | | | | |  | |
|  | Blood sugar measurement time M4 year moon M4a Fasting blood glucose mmol/L M4b HbA1c mmol/L | | | | | | | |  | |
|  | Blood sugar measurement time M5 year moon M5a Fasting Blood Glucose mmol/L M5b HbA1c mmol/L | | | | | | | |  | |
|  | Blood sugar measurement time M6 year moon M6a fasting blood glucose mmol/L M6b HbA1c mmol/L | | | | | | | |  | |
|  | Blood sugar measurement time M7 year moon M7a fasting blood glucose mmol/L M7b HbA1c mmol/L | | | | | | | |  | |
|  | Blood pressure measurement time M8 year moon M8a high pressure MmHg M8b low pressure mmHg | | | | | | | |  | |
|  | Blood lipid measurement time M9 year moon M9a total cholesterol M9b triglyceride mmol/L | | | | | | | |  | |
|  | M9c low density lipoprotein Mmol/L M9d high density lipoprotein mmol/L | | | | | | | |  | |
