# Supplementary figures and images for: Healthcare utilization and expenditures among adults with type 2 diabetes mellitus and comorbid psychological distress
Source: Front Endocrinol (Lausanne). 2026 Jan 29;17:1702996. doi: 10.3389/fendo.2026.1702996 (PMC12893985; doi:10.3389/fendo.2026.1702996)

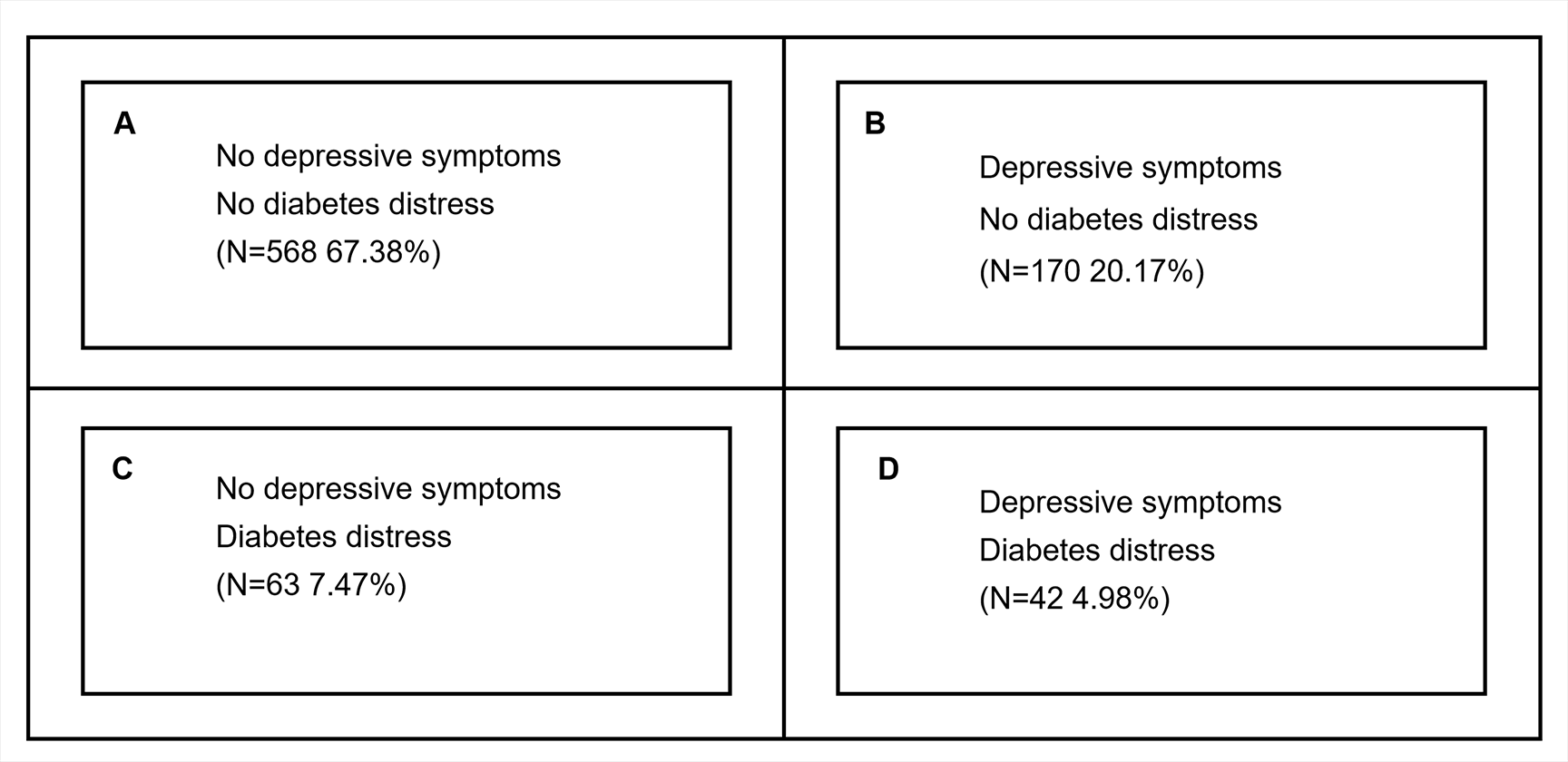

Supplement: Supplementary file 2 [file Image1.tif]
