# Supplementary material for: Healthcare utilization and expenditures among adults with type 2 diabetes mellitus and comorbid psychological distress
Source: Front Endocrinol (Lausanne). 2026 Jan 29;17:1702996. doi: 10.3389/fendo.2026.1702996 (PMC12893985; doi:10.3389/fendo.2026.1702996)
Supplement: Supplementary file 3 [file Table1.docx]

Additional Table 1. Basic characteristics in adults with type 2 diabetes mellitus (N=843)

| **Variable** | **Diabetes mellitus**  **（N=568）** | **Comorbid diabetes distress**  **（N=63）** | **Comorbid** **depression symptoms**  **（N=170）** | **Comorbid diabetes distress**  **and depression symptoms**  **（N=42）** | **P** |
| --- | --- | --- | --- | --- | --- |
|  | **N(%)** | **N(%)** | **N(%)** | **N(%)** |  |
| ***Gender*** |  |  |  |  |  |
| Male | 208(36.62) | 15(23.81) | 50(29.41) | 9(21.43) | 0.027* |
| Female | 360(63.38) | 48(76.19) | 120(70.59) | 33(78.57) |  |
| ***Age (year)*** |  |  |  |  |  |
| <60 | 140(24.65) | 19(30.16) | 31(18.24) | 9(21.43) | 0.049* |
| 60-70 | 205(36.09) | 22(34.92) | 78(45.88) | 23(54.76) |  |
| > 70 | 223(39.26) | 22(34.92) | 61(35.88) | 10(23.81) |  |
| ***Education*** |  |  |  |  |  |
| Illiterate | 210(36.97) | 20(31.75) | 81(47.65) | 21(50.00) | 0.005† |
| Primary | 161(28.35) | 22(34.92) | 55(32.35) | 14(33.33) |  |
| Secondary | 143(25.18) | 19(30.16) | 24(14.12) | 4(9.52) |  |
| Post-Secondary | 54(9.51) | 2(3.17) | 10(5.88) | 3(7.14) |  |
| ***Marital status*** |  |  |  |  |  |
| Married | 480(84.51) | 53(84.13) | 128(75.29) | 34(80.95) | 0.049* |
| Unmarried^a^ | 88(5.49) | 10(15.87) | 42(24.71) | 8(19.05) |  |
| ***Duration of diabetes*** |  |  |  |  |  |
| ≤4 | 154(27.11) | 13(20.63) | 40(23.53) | 4(9.52) | 0.003† |
| 5-7 | 180(31.69) | 24(38.10) | 41(24.12) | 14(33.33) |  |
| 8-11 | 124(21.83) | 16(25.40) | 37(21.76) | 7(16.67) |  |
| ＞11 | 110(19.37) | 10(15.87) | 52(30.59) | 17(40.48) |  |
| ***Comorbidities***^b^ |  |  |  |  |  |
| Cardiovascular diseases | 87(15.32) | 9(14.29) | 33(19.41) | 6(14.29) | 0.594 |
| Hypertension | 323(56.87) | 43(68.25) | 98(57.65) | 26(61.90) | 0.349 |
| Cerebrovascular diseases | 39(6.87) | 6(9.52) | 22(12.94) | 5(11.90) | 0.074 |
| Other diseases | 278(48.94) | 33(52.38) | 93(54.71) | 28(66.67) | 0.109 |
| ***Baseline FPG (mmol/L)*** |  |  |  |  |  |
| FPG<7.0 | 212(37.32) | 26(41.27) | 65(38.24) | 16(38.10) | 0.942 |
| FPG≥7.0 | 356(62.68) | 37(58.73) | 105(61.76) | 26(61.90) |  |
| ***DDS scores*** | 1.33±0.28 | 2.18±0.26 | 1.48±0.30 | 2.49±0.52 | < 0.001‡ |
| ***CES-D scores*** | 3.45±2.94 | 4.40±3.27 | 14.15±3.72 | 17.29±5.31 | < 0.001‡ |

Abbreviation：DDS, Diabetes Distress Scale; CES-D, Center for Epidemiologic Studies Depression Scale; FPG, fasting plasma glucose;

^a^unmarried included separated ,divorced,widowed or never married.

^b^Comorbidities included cardiovascular diseases, hypertension, cerebrovascular diseases,and other chronic conditions.

*P < 0.05; † P < 0.01；‡ P < 0.001

Additional Table 2.Basic characteristics of healthcare utilization and expenditures

|  | **Annual number of outpatient visits**  **[M(QR)]** | | **Utilization of inpatient services**  **[N(%)]** | **Outpatient costs**  **[M(QR),￥]** | **Drug costs**  **[M(QR),￥]** | **Inpatient costs**  **[M(QR),￥]** | **Total medical expenditures [M(QR),￥]** |
| --- | --- | --- | --- | --- | --- | --- | --- |
| **Diabetes mellitus**  **（N=568）** | 0±2 | 148(26.06) | | 600±1200 | 1000±1700 | 5000±9000 | 3000±6500 |
| **Comorbid** **diabetes distress**  **（N=63）** | 0±3 | 20(31.75) | | 1500±1750 | 1200±1800 | 9000±9500 | 5000±7900 |
| **Comorbid depression** **symptoms**  **（N=170）** | 0±3 | 63(37.06) | | 800±1700 | 1000±2200 | 7000±11500 | 4150±8070 |
| **Comorbid diabetes distress**  **and depressive symptoms**  **（N=42）** | 2±4 | 18(42.86) | | 1000±1500 | 1100±2200 | 6000±7000 | 5000±6240 |
| **χ²** | 10.72* | 11.66† | | 8.81* | 4.58 | 3.67 | 13.19† |
| ***p-value*** | 0.013 | 0.009 | | 0.032 | 0.206 | 0.300 | 0.004 |

Additional Table 3. Regression analysis of psychological health on healthcare utilization in adults with type 2 diabetes mellitus

|  | Annual number of outpatient visits | | Utilization of inpatient services | |
| --- | --- | --- | --- | --- |
| Variable | Model 1 | Model 2 | Model 1 | Model 2 |
|  | IRR(95%CI) | IRR(SE) | OR(SE) | OR(SE) |
| ***Psychological health*** (ref: Diabetes mellitus ) |  |  |  |  |
| Comorbid diabetes distress | 1.39(0.79-2.45) | 1.40(0.79-2.45) | 1.32(0.75-2.32) | 1.32(0.74-2.37) |
| Comorbid depressive symptoms | 1.47(1.01-2.13)* | 1.52(1.05-2.21)* | 1.67(1.16-2.40)† | 1.50(1.02-2.19)* |
| Comorbid diabetes distress and depressive symptoms | 2.03(1.04-3.98)* | 2.31(1.16-4.59)* | 2.13(1.12-4.03)* | 1.88(0.96-3.66) |
| ***Age*** |  | 0.98(0.96-1.00)* |  | 1.02(1.00-1.04) |
| ***Gender*** |  |  |  |  |
| Female (ref: Male) |  | 0.84(0.58-1.20) |  | 0.71(0.49-1.03) |
| ***Education*** (ref: lliterate) |  |  |  |  |
| Primary |  | 0.87(0.59-1.30) |  | 1.05(0.72-1.54) |
| Secondary |  | 1.00(0.63-1.59) |  | 0.89(0.56-1.43) |
| Post-Secondary |  | 0.91(0.47-1.79) |  | 0.69(0.34-1.38) |
| ***Marital status*** |  |  |  |  |
| Married (ref: Unmarried) |  | 1.51(0.98-2.32) |  | 1.18(0.78-1.81) |
| ***Duration of diabetes*** |  | 1.02(0.99-1.05) |  | 1.04(1.01-1.06)† |
| ***Cardiovascular disease*** |  |  |  |  |
| With (ref: Without) |  | 1.75(1.18-2.60)† |  | 1.20(0.79-1.80) |
| ***Hypertension*** |  |  |  |  |
| With (ref: Without) |  | 1.26(0.92-1.73) |  | 1.12(0.81-1.55) |
| ***Cerebrovascular disease*** |  |  |  |  |
| With (ref: Without) |  | 1.90(1.13-3.19)* |  | 2.00(1.20-3.34)† |
| ***Other chronic diseases*** |  |  |  |  |
| With (ref: Without) |  | 1.66(1.24-2.23)† |  | 1.72(1.25-2.36)† |
| ***Baseline FPG*** |  | 0.98(0.92-1.04) |  | 0.99(0.93-1.05) |

Note: all coefficients are unstandardized. Gender was coded as male=0, female=1. Education was coded as illiterate=0, primary=1, secondary=2, post-secondary=3. Marital status was coded as unmarried=0, married=1. Cardiovascular disease was coded as without=0, with=1. Hypertension was coded as without=0, with=1. Cerebrovascular disease was coded as without=0, with=1.Other chronic conditions was coded as without=0, with=1.

Abbreviation：DDS, Diabetes Distress Scale; CES-D, Center for Epidemiologic Studies Depression Scale; FPG, fasting plasma glucose;

*P < 0.05; † P < 0.01；‡ P < 0.001

Additional Table 4. Regression analysis of psychological health on healthcare expenditures in adults with type 2 diabetes mellitus

| Variable | Outpatient costs | | Inpatient costs | | Drug costs | | Total medical expenditures | |
| --- | --- | --- | --- | --- | --- | --- | --- | --- |
|  | Model 1 | Model 2 | Model 3 | Model 4 | Model 5 | Model 6 | Model 7 | Model 8 |
|  | β(95%CI) | β(95%CI) | β(95%CI) | β(95%CI) | β(95%CI) | β(95%CI) | β(95%CI) | β(95%CI) |
| ***Psychological health***  (ref: Diabetes mellitus ) |  |  |  |  |  |  |  |  |
| Comorbid diabetes distress | 0.61(-0.03-1.24) | 0.63(-0.01-1.28) | 0.54(-0.11-1.19) | 0.67(0.01-1.33)* | 0.74(0.03-1.45)* | 0.72(-0.02-1.46) | 0.75(0.21-1.28)† | 0.76(0.22-1.31)† |
| Comorbid depressive symptoms | 0.13(-0.23-0.49) | 0.15(-0.22-0.52) | 0.30(-0.12-0.71) | 0.38(-0.04-0.80) | 0.15(-0.27-0.57) | 0.16(-0.28-0.59) | 0.36(0.02-0.69)* | 0.33(-0.01-0.67) |
| Comorbid diabetes distress and depressive symptoms | 0.63(0.03-1.22)* | 0.64(0.04-1.25)* | 0.28(-0.40-0.96) | 0.31(-0.38-1.00) | 0.35(-0.38-1.09) | 0.38(-0.38-1.14) | 0.68(0.12-1.24)* | 0.69(0.12-1.26)* |
| ***Age*** |  | 0.01(-0.02-0.02) |  | 0.01(-0.02-0.03) |  | 0.01(-0.02-0.03) |  | 0.01(-0.01-0.03) |
| ***Gender*** |  |  |  |  |  |  |  |  |
| Female (ref: Male) |  | -0.06(-0.43-0.31) |  | -0.38(-0.78-0.02) |  | 0.10(-0.36-0.56) |  | -0.17(-0.51-0.16) |
| ***Education*** (ref: illiterate) |  |  |  |  |  |  |  |  |
| Primary |  | -0.15(-0.54-0.24) |  | 0.29(-0.14-0.72) |  | 0.06(-0.43-0.54) |  | 0.18(-0.16-0.53) |
| Secondary |  | 0.39(-0.07-0.84) |  | -0.07(-0.61-0.47) |  | 0.19(-0.36-0.75) |  | 0.04(-0.38-0.46) |
| Post-Secondary |  | 0.08(-0.56-0.71) |  | 0.48(-0.35-1.32) |  | 0.07(-0.68-0.82) |  | 0.06(-0.55-0.67) |
| ***Marital status*** |  |  |  |  |  |  |  |  |
| Married (ref: Unmarried) |  | 0.04(-0.39-0.46) |  | 0.12(-0.35-0.59) |  | 0.20(-0.33-0.73) |  | 0.11(-0.26-0.49) |
| ***Duration of diabetes*** |  | 0.02(-0.01-0.05) |  | -0.01(-0.04-0.02) |  | 0.03(-0.01-0.06) |  | 0.02(-0.01-0.04) |
| ***Cardiovascular disease*** |  |  |  |  |  |  |  |  |
| With (ref: Without) |  | 0.24(-0.14-0.62) |  | -0.03(-0.48-0.41) |  | -0.22(-0.70-0.26) |  | 0.10(-0.26-0.45) |
| ***Hypertension*** |  |  |  |  |  |  |  |  |
| With (ref: Without) |  | 0.05(-0.27-0.38) |  | -0.27(-0.65-0.12) |  | 0.18(-0.21-0.57) |  | 0.01(-0.29-0.30) |
| ***Cerebrovascular disease*** |  |  |  |  |  |  |  |  |
| With (ref: Without) |  | 0.11(-0.36-0.58) |  | 0.03(-0.48-0.54) |  | -0.24(-0.79-0.32) |  | 0.23(-0.21-0.67) |
| ***Other chronic diseases*** |  |  |  |  |  |  |  |  |
| With (ref: Without) |  | 0.05(-0.27-0.37) |  | -0.32(-0.68-0.04) |  | -0.08(-0.46-0.30) |  | -0.01(-0.29-0.27) |
| ***Baseline FPG*** |  | -0.05(-0.11-0.01) |  | -0.02(-0.09-0.04) |  | -0.01(-0.08-0.06) |  | -0.02(-0.07-0.04) |

Note:*P < 0.05; † P < 0.01；‡ P < 0.001
